# Potential Coverage of the 4CMenB Vaccine against Invasive Serogroup B *Neisseria meningitidis* Isolated from 2009 to 2013 in the Republic of Ireland

**DOI:** 10.1128/mSphere.00196-18

**Published:** 2018-08-22

**Authors:** Robert M. Mulhall, Desiree Bennett, Robert Cunney, Ray Borrow, Jay Lucidarme, Jamie Findlow, Keith A. Jolley, James Bray, Martin C. J. Maiden, Monica Moschioni, Laura Serino, Maria Stella, Duccio Medini

**Affiliations:** a Irish Meningitis and Sepsis Laboratory, Temple Street Children’s University Hospital, Dublin, Ireland; b Health Protection Surveillance Centre, Dublin, Ireland; c Meningococcal Reference Unit, Public Health England, Manchester, United Kingdom; d Department of Zoology, University of Oxford, Oxford, United Kingdom; e GSK Vaccines, Siena, Italy; University of Maryland School of Medicine.

**Keywords:** 4CMenB, MATS, MenB, NHBA, NadA, *Neisseria meningitidis*, vaccine, fHbp, *porA*

## Abstract

Neisseria meningitidis is a common cause of bacterial meningitis in children and young adults worldwide. The 4CMenB vaccine (Bexsero), developed to combat meningococcal serogroup B (MenB) disease, contains subcapsular antigens that may induce immunity against strains of N. meningitidis, regardless of serogroup. Owing to differential levels of expression and peptide diversity in vaccine antigens across meningococcal strains, the meningococcal antigen typing system (MATS) was developed to estimate the potential MenB strain coverage of 4CMenB. Prior to introducing the 4CMenB vaccine into routine use, we sought to estimate the potential 4CMenB coverage against invasive MenB strains isolated in the Republic of Ireland (RoI) over four consecutive epidemiological years. MATS was applied to a panel of 105 invasive MenB strains isolated during July 2009 to June 2013. Sequence data characterizing the multilocus sequence typing (MLST) alleles and the major 4CMenB target peptides were extracted from isolate genome sequence data, hosted in the Bacterial Isolate Sequencing database (BIGSdb). MATS data indicated that 4CMenB may induce protective immunity against 69.5% (95% confidence interval [CI_95%_], 64.8% to 84.8%) of circulating MenB strains. Estimated coverage was highest against the most prevalent disease-causing lineage, cc41/44, where the most frequently observed sequence types, ST-154 and ST-41 (21% of isolates, collectively), were typically covered by three antigens. No significant temporal trends were observed. Overall, these data provide a baseline of strain coverage prior to the introduction of 4CMenB and indicate that a decrease in invasive meningococcal disease (IMD) is predicted following the introduction of 4CMenB into the routine infant immunization schedule in the RoI.

**IMPORTANCE** The meningococcal antigen typing system (MATS) is an enzyme-linked immunosorbent assay (ELISA) that measures both the levels of expression and the immune reactivity of the three recombinant 4CMenB antigens. Together with PorA variable-region sequence data, this system provides an estimation of how susceptible MenB isolates are to killing by 4CMenB vaccine-induced antibodies. Assays based on subcapsular antigen phenotype analyses, such as MATS, are important in situations where conventional vaccine coverage estimations are not possible. Subcapsular antigens are typically highly diverse across strains, and vaccine coverage estimations would require unfeasibly large efficacy trials and screening of an exhaustive strain panel for antibody functional activity. Here, MATS was applied to all invasive meningococcal serogroup B (MenB) strains isolated over four consecutive epidemiological years (*n* = 105) and predicted reasonably high 4CMenB vaccine coverage in the Republic of Ireland.

## Abstract (precis)

The meningococcal antigen typing system (MATS) is an enzyme-linked immunosorbent assay (ELISA) that measures both the levels of expression and the immune reactivity of the three recombinant 4CMenB antigens. Together with PorA variable-region sequence data, this system provides an estimation of how susceptible MenB isolates are to killing by 4CMenB vaccine-induced antibodies. Assays based on subcapsular antigen phenotype analyses, such as MATS, are important in situations where conventional vaccine coverage estimations are not possible. Subcapsular antigens are typically highly diverse across strains, and vaccine coverage estimations would require unfeasibly large efficacy trials and screening of an exhaustive strain panel for antibody functional activity. Here, MATS was applied to all invasive meningococcal serogroup B (MenB) strains isolated over four consecutive epidemiological years (*n* = 105) and predicted reasonably high 4CMenB vaccine coverage in the Republic of Ireland.

## INTRODUCTION

The relatively low incidence of invasive meningococcal disease (IMD) currently experienced in Europe (1) is due in part to the routine use of capsular polysaccharide conjugate vaccines, which have reduced disease incidence from meningococci expressing capsular types C, W, and Y (2, 3). In common with many other European countries, the population of the Republic of Ireland (RoI) predominantly experiences serogroup B IMD (1, 4), with laboratory-confirmed incidence rates of 1.37 cases per 100,000 population in 2013 to 2014, 1.24 in 2014 to 2015, and 0.99 in 2015 to 2016 (http://www.hpsc.ie). Meningococcal disease incidence peaks within the first year of life and then declines until the middle to late teenage years, when a second, smaller peak is observed. Children under 5 years of age suffer the highest disease burden (http://www.hpsc.ie). The majority of meningococcal disease observed is sporadic and is caused by meningococci with multiple distinct genotypes as defined by multilocus sequence typing (MLST) (5).

The MenB polysaccharide is poorly immunogenic, and possibly autoantigenic, and so a MenB polysaccharide vaccine is considered unfeasible (6, 7). Consequently, subcapsular antigens have been exploited instead. Several outer membrane vesicle (OMV) vaccines have been demonstrated to be safe and effective, protecting against disease caused by homologous meningococci (8–11). These vaccines elicit mostly strain-specific antibody responses; poor cross-reactive responses to heterologous strains are observed in adults, and none are observed in infants (12, 13). OMV vaccines are therefore limited to combating occurrences of clonal expansion and are unsuitable in controlling heterologous endemic strains.

A four-component vaccine, 4CMenB (Bexsero) (14), is currently licensed for use in over 30 countries worldwide and was introduced into the schedule of routine immunization of infants (at 2, 4, and 12 months of age) in the RoI in December 2016 and in the United Kingdom in September 2015 (15, 16). The major antigens include recombinant forms of neisserial adhesin A (NadA) peptide 3 (variant NadA-2/3), factor-H binding protein (fHbp) peptide 1 (variant 1), *Neisseria* heparin-binding antigen (NHBA) peptide 2, and New Zealand strain outer membrane vesicles (NZ OMV) containing the PorA protein with the variable-region 2 (VR2) peptide P1.4 (10, 14).

4CMenB has demonstrated effective protection against diverse MenB in the United Kingdom, with 83% (95% confidence interval [CI_95%_], 24% to 95%) two-dose vaccine effectiveness against all MenB cases in the first 10 months of the infant national immunization program (17).

Vaccine effectiveness depends on host (immune response) factors and pathogen (strain coverage) factors. Polysaccharide and conjugate vaccine immunogenicity is assessed by the serum bactericidal antibody (SBA) assay, which measures complement-mediated killing for representative strains expressing different serogroups (18–22). Strain coverage for vaccines which target the polysaccharide can be estimated by observing the serogroup frequencies of invasive meningococcal populations. This approach is not practical for subcapsular protein antigens, which are likely to vary both in their levels of expression and in their peptide sequences across the many different endemic disease-associated meningococcal strains (23, 24). MLST-based lineage frequencies cannot be used either, due to the dynamic and highly recombining population structure of meningococcal species (25).

As an alternative, the meningococcal antigen typing system (MATS), which combines genotyping for PorA and a sandwich enzyme-linked immunosorbent assay (ELISA), was developed to measure the level of target antigen expression and ability of 4CMenB-induced antibodies to recognize the vaccine antigens on individual invasive MenB isolates (26). This capacity of vaccine-induced antibodies to recognize their respective vaccine targets is referred to as the relative potency (RP). Test isolates are considered covered by MATS if the RP level is greater than the positive bactericidal threshold (PBT) for at least one vaccine antigen (fHbp, NHBA, or NadA) or if they harbor PorA VR2 peptide P1.4 (27) or both. MATS-PBT values correlate vaccine-induced SBA titers with bactericidal killing and were established using postvaccination pooled sera from infants after a fourth dose of 4CMenB at 12 months (26). These experiments link MATS-PBTs and the meningococcal gold standard correlate of protection (SBA titer of ≥4), which is the accepted surrogate to assess vaccine effectiveness in the absence of protection studies (18, 22, 28). To ensure consistency of MATS data, interlaboratory standardization studies have been conducted in National Reference Laboratories for meningococcal disease (29), including Public Health England’s meningococcal reference unit (PHE-MRU, Manchester, United Kingdom) and the U.S. Centers for Disease Control and Prevention (CDC, Atlanta, GE).

The aim of this study was to evaluate the potential coverage, as defined by MATS, of MenB IMD-associated isolates collected during the 2009-2010 to 2012-2013 epidemiological years in the RoI and from different age groups. We aimed to describe 4CMenB target peptide diversity and the relationship with meningococcal lineages, as defined by MLST, before the introduction of the vaccine.

## RESULTS

### Distribution of isolates by clonal complex and age.

The 105 isolates represented 53 different sequence types (STs) and 13 clonal complexes (cc). The most frequently observed clonal complexes were cc41/44 (*n* = 45, 43%), cc269 (*n* = 23, 22%), cc213 (*n* = 5, 5%), cc32 (*n* = 5, 5%), cc162 (*n* = 4, 4%), and cc461 (*n* = 3, 3%) (Table 1). Two isolates each of cc18, cc35, and cc60 (*n* = 2, 2%) and a single isolate each of cc103, cc1157, cc254, and cc22 were also observed. A further 10 STs (10%) were unassigned to a clonal complex (Table 1).

**TABLE 1:** Prevalence of the most frequently observed clonal complexes and sequence types by epidemiological year

Clonal complex	Epidemiological yr				Total
	2009–2010	2010–2011	2011–2012	2012–2013	
cc41/44 (*n* = 45)					
Total	15	13	6	11	45
154	5	4	2	3	14
41	4	1	2		7
1194		2	1	2	5
1403			1	1	2
7670	1	1			2
1475				2	2
Others^a^	5	5		3	13
					
cc269 (*n* = 23)					
Total	11	4	3	5	23
1163	2	1		2	5
269	2	1	1	1	5
479	2		1	1	4
7854	2		1		3
10544		1		1	2
Others^b^	3	1			4
					
cc213 (*n* = 5)					
Total	1	1	1	2	5
575		1	1	1	3
Others^c^	1			1	2
					
cc32 (*n* = 5)					
Total	2	1	1	1	5
32		1	1		2
6083	1			1	2
33	1				1
					
cc162 (*n* = 5)					
Total	3		1		4
162	3		1		4
					
Unassigned (*n* = 10)^d^					
Total	2	2	3	3	10
1193	1		1	2	4
—^e^	1		1		2
Others		2	1	1	4

^a^ Other cc41/44 sequence types observed once only were ST-8885, ST-10543, ST-9574, ST-1414, ST-8384, ST-10985, ST-8951, ST-40, ST-10132, ST-6697, ST-340, ST-274, and ST-3818.

^b^ Other cc269 sequence types observed once only were ST-1273, ST-1161, ST-275, and ST-1214.

^c^ Other cc213 sequence types observed once only were ST-10133 and ST-9577.

^d^ Sequence types that were not assigned to a clonal complex (*n* = 10) included ST-1193 (*n* = 4), an undefined sequence type, and a single example each of ST-1575, ST-8950, ST-7143, and ST-4388.

^e^ —, undefined sequence type.

Together, ST-154 (*n* = 14), ST-41 (*n* = 7), and ST-1194 (*n* = 5) accounted for 58% (*n* = 26/45) of the cc41/44 isolates, while the remaining 19 isolates were representative of 16 different STs, 13 of which were observed once each (Table 1). A more even distribution of ST was observed among the cc269 isolates (*n* = 23), which included ST-1163 (*n* = 5), ST-269 (*n* = 5), ST-479 (*n* = 4), ST-7854 (*n* = 3), and ST-10544 (*n* = 2) and four STs observed once only.

The most prevalent lineages, cc41/44 and cc269, were more likely to cause disease in children under 5 years of age and in children over 1 year of age, respectively, with observed odds ratios (OR) of 2.1 (CI_95%_, 0.91 to 4.8, *P* = 0.08) and 4.1 (CI_95%_, 1.28 to 13.11, *P* < 0.02), respectively (data not shown).

### Coverage estimates by age and epidemiological year.

**FIG 1:**
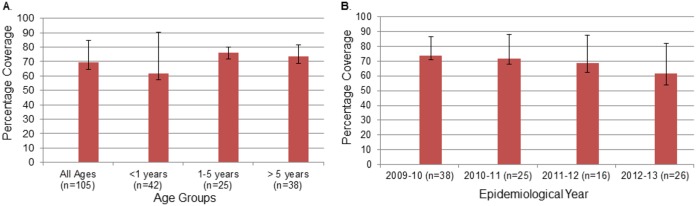
Potential coverage of the 4CMenB vaccine in the Republic of Ireland by age (A) and by epidemiological year (B). Data are based on 105 serogroup B invasive Neisseria meningitidis isolates collected during the 2009-2010 to 2012-2013 epidemiological years.

The overall coverage estimate for the 4CMenB vaccine was 69.5% (CI_95%_, 64.8% to 84.8%) (Fig. 1A). Point estimates of predicted strain coverage by year were similar across the different age groups, with the highest anticipated for children 1 to 5 years of age (76%; CI_95%_, 72% to 80%) and lowest for those under 1 year of age (61.9%; CI_95%_, 57.1% to 90.5%) (Fig. 1A). A 2-sided chi-square test was used to test the differences across years in the predicted MATS coverage, and no statistical significant difference was observed (*P* value for the age groups overall, 0.376; one-to-one-comparison *P* values, 0.358, 0.376, and 1 for children under 1 year of age, 1 to 5 years of age, and more than 5 years of age, respectively). The 2-sided chi-square test was also used to test the differences across epidemiological years in the predicted MATS coverage from 2009 to 2013 (Fig. 1B). No statistical significant difference was observed (overall, *P* = 0.76; one-to-one comparisons, *P* > 0.45 to *P* ≤ 1). Point estimates varied from 73.7% (CI_95%_, 71.1% to 86.8%) in 2009 to 2010 to 61.5% (CI_95%_, 53.8% to 76.9%) in 2012 to 2013 (Fig. 1B).

### Coverage by antigen and by antigen combinations.

**FIG 2:**
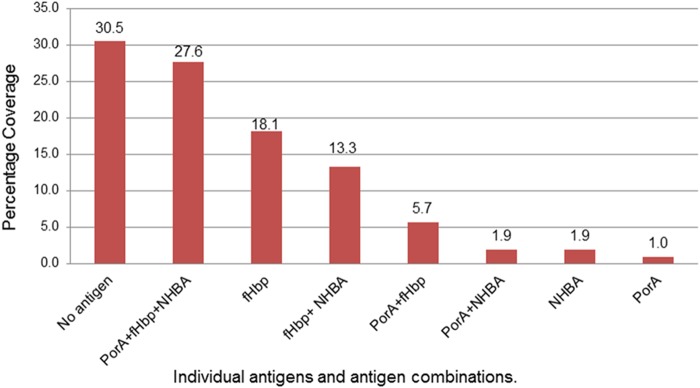
MATS-predicted coverage by specific antigen and by antigen combinations (*n* = 105).

MATS results revealed that no isolate was predicted to be covered by all four vaccine antigens (Fig. 2). Thirty-two isolates (31%) were not expected to be covered. The proportion of isolates covered by a single antigen was 21% (*n* = 22/105), and the proportion of isolates covered by two antigens was also 21% (*n* = 22/105), while 28% of isolates were covered by three antigens (*n* = 29/105). Strains responsible for invasive disease in subjects under 1 year of age were more likely to harbor 3 MATS-positive peptides than the strains from those over 1 year of age (*P* < 0.02).

The proportions of isolate coverage by individual target antigens were 36% (*n* = 38) for PorA, 64.8% (*n* = 68; CI_95%_, 55.2% to 72.4%) for fHbp, and 44.8% (*n* = 47; CI_95%_, 27.6% to 64.8%) for NHBA. NadA was present as a functional gene in ST-32 complex isolates only (*n* = 5), with RP values of 0.000 (*n* = 2), 0.001 (*n* = 2), and 0.005 (*n* = 1). These values were below the MATS-PBT of 0.009 for NadA.

### Coverage by the most prevalent clonal complexes and sequence types.

Coverage estimates by clonal complex were as follows: 97.8% for cc41/44 (*n* = 44/45; CI_95%_, 97.8% to 97.88%) and 65.2% for cc269 (*n* = 15/23; CI_95%_, 60.9% to 69.6%). Estimates of the coverage of cc269 isolates varied nonsignificantly with age and were lowest in the group of those under 1 year of age (50%; *n* = 2/4) and highest in the group of those over 5 years of age (80%, *n* = 8/10). The next most prevalent complexes were cc32 and cc213, where coverage was predicted to be 100% (*n* = 5/5; CI_95%_, 100% to 100%) and 0% (n = 0/5 CI_95%_, 0% to 20%), respectively (data not shown). The overall coverage estimated for all other clonal complex isolates was 33.3% (*n* = 9/27; CI_95%_, 18.5% to 85.2%) (data not shown).

Coverage was estimated at 100% (CI_95%_, 100% to 100%) for both ST-154 (*n* = 14) and ST-41 (*n* = 7), the two most prevalent STs isolated during the epidemiological period beginning in July 2009 and ending in June 2013 (*n* = 21/105; 20.0%).

### 4CMenB peptide prevalence.

Fifteen unique PorA VR2 peptides were observed among 104 of the 105 isolates harboring the *porA* gene; the *porA* gene was undetected in a single isolate. Seven VR2 peptides were observed once only. Three PorA VR2 peptides, P1.4 (the vaccine type, *n* = 38, 36%), P1.9 (*n* = 15, 14%), and P1.14 (*n* = 14, 13%), collectively accounted for 64% of all VR2 peptides observed. Genes for the P1.4 vaccine peptide were predominantly (*n* = 35/38, 92%) possessed by cc41/44 isolates; 50% (*n* = 2/4) of the cc162 isolates possessed genes for the P1.4 vaccine peptide, and a single isolate was not assigned to a complex.

The relationships among clonal complexes, the most frequent NHBA peptides, and MATS-predicted coverage for the NHBA antigen are shown in Table 2. Twenty-two different NHBA peptides were identified, with 10 occurring once and 5 occurring twice. The most frequently observed NHBA peptides were as follows: peptide 2 (the vaccine type, *n* = 31, 29.5%), peptide 21 (*n* = 17, 16.2%), peptides 17 and 20 (*n* = 11, 10.5%), peptide 607 (*n* = 5, 4.8%) and peptides 18 and 24 (*n* = 4, 3.8%) (Table 2; see also Fig. S1 in the supplemental material). NHBA peptide 2 was identified significantly more often in those ≤5 years of age (*P* < 0.03) and was exclusively found in cc41/44 isolates. cc41/44 isolates harbored nine other peptides, which included peptide 607 (*n* = 5), peptide 160 (*n* = 2), and seven peptides which were observed once only. The next most commonly observed NHBA peptides were identified mostly in cc269 isolates; peptide 21 (*n* = 14/23) and peptide 17 (*n* = 7/23) together accounted for the majority of cc269 NHBA peptides (*n* = 21/23). Peptide 20 was found in all cc162 isolates (*n* = 4) and in three other complexes.

10.1128/mSphere.00196-18.1 FIG S1  Relative potency values for the most frequently observed NHBA peptides. Download FIG S1, PDF file, 0.02 MB. Copyright © 2018 Mulhall et al. 2018 Mulhall et al. This content is distributed under the terms of the Creative Commons Attribution 4.0 International license.

**TABLE 2:** Relationship between clonal complex, NHBA peptide, and MATS-predicted coverage for 105 disease-associated serogroup B isolates

NHBA peptide	Predicted coverage from MATS-NHBA relative potency values^a^																% NHBA peptide prevalence
	cc41/44 (*n* = 45)		cc269 (*n* = 23)		cc213 (*n* = 5)		cc32 (*n* = 5)		cc162 (*n* = 4)		cc35 (*n* = 2)		Others		Overall (*n* = 105)		
	Total	Cov.	Total	Cov.	Total	Cov.	Total	Cov.	Total	Cov.	Total	Cov.	Total	Cov.	Total	Cov.	
2	31	30 (97)													31	30 (97)	29.5
21			14	7 (50)							2	0	1	0	17	7 (41)	16.2
17			7	0									4	0	11	0	10.5
20							1	0	4	1 (25)			6	1 (17)	11	2 (18)	10.5
18					4	0									4	0	3.8
24	1	0											3	0	4	0	3.8
607	5	4 (80)													5	4 (80)	4.8
Others	8	2 (25)	2	0	1	0	4	2 (50)					7	0	22	4 (18)	21

^a^ Total, total number of isolates tested; Cov., total number (percent) of isolates covered.

All isolates possessed complete *fHbp* genes. Twenty-three different fHbp peptides were identified, 12 were observed once and 3 twice. The most frequently identified peptides were as follows: fHbp peptide 4 (*n* = 30 28.6%), fHbp peptide 15 (*n* = 15 14.3%), fHbp peptide 14 (*n* = 13 12.4%), fHbp peptide 13 (*n* = 11 10.5%), fHbp peptide 19 (*n* = 7 6.7%), and fHbp peptide 16 (*n* = 6 5.7%) (Table 3; see also Fig. S2). Peptide 4 was found exclusively in the cc41/44 isolates (*n* = 30), which also harbored peptide 14 (*n* = 13) and a single example each of peptides 19 and 110. The estimated fHbp coverage for the cc41/44 isolates was 93.3% (*n* = 42/45).

10.1128/mSphere.00196-18.2 FIG S2  Relative potency values for the most frequently observed FHbp peptides. Download FIG S2, PDF file, 0.02 MB. Copyright © 2018 Mulhall et al. 2018 Mulhall et al. This content is distributed under the terms of the Creative Commons Attribution 4.0 International license.

**TABLE 3:** Relationship between clonal complex, fHbp peptides, and MATS-predicted coverage for 105 disease-associated serogroup B isolates

fHbp peptide	Predicted coverage from MATS-fHbp relative potency values^a^																% fHbp peptide prevalence
	cc41/44 (*n* = 45)		cc269 (*n* = 23)		cc213 (*n* = 5)		cc32 (*n* = 5)		cc162 (*n* = 4)		cc35 (*n* = 2)		Others		Overall (*n* = 105)		
	Total	Cov.	Total	Cov.	Total	Cov.	Total	Cov.	Total	Cov.	Total	Cov.	Total	Cov.	Total	Cov.	
4^b^	30	30 (100)													30	30 (100)	29
15^b^			15	15 (100)											15	15 (100)	14
14^b^	13	11 (85)													13	11 (85)	12
13^b^			1	0	2	0	1	1 (100)					7	2 (29)	11	3 (27)	11
19	1	0	6	0											7	0	7
16											2	0	4	1 (100)	6	1 (17)	6
1^b^							3	3 (100)							3	3 (100)	3
Others	1	1 (100)	1	0	3	0	1	1 (100)	4	1 (25)			10	3 (30)	20	6 (30)	19

^a^ Total, total number of isolates tested; Cov., total number (percent) of isolates covered.

^b^ fHbp peptide variant from family 1 (Novartis nomenclature).

fHbp peptide variation can be classified into three families (30). The prevalences of fHbp variants in this study and their MATS-predicted coverage were as follows: family 1 variants, 75.2% (*n* = 79/105), among which 86.1% were covered by fHbp MATS (*n* = 68/79); family 2 variants, 18.1% (*n* = 19/105) of which showed no coverage (*n* = 0/19); family 3 variants, 6.7% (*n* = 7/105) of which were predicted to have no coverage.

Only cc32 isolates (*n* = 5/5) harbored intact *nadA* genes potentially encoding functional proteins (peptide 1 [*n* = 4] and peptide 100 [*n* = 1]) but failed to generate an RP value for NadA above the PBT. These RP values may be underestimated since NadA expression is repressed *in vitro* but not *in vivo* (31) and may contribute to *in vivo* killing. In any case, the overall coverage estimate would not change as these five isolates were anticipated to be covered by fHbp. All cc213 isolates possessed NadA-4/5 alleles, which were frameshifted at an intragenic homopolymer and, if in-frame, would not express cross-reactive peptides. Three further contained atypical *nadA* alleles not anticipated to express functional forms (alleles ml1157 [*n* = 2] and ml103 [*n* = 1] and a single unassigned ST [incomplete *fumC* sequence]).

### 4CMenB peptide prevalence among the most prevalent sequence types.

The most prevalent sequence types, ST-154 (*n* = 14) and ST-41 (*n* = 7), were anticipated to be covered by fHbp. NHBA peptide 2 was expressed in 92.9% of ST-154 isolates (*n* = 13/14) and 85.7% of ST-41 isolates (*n* = 6/7). These two STs were more likely to be associated with IMD in those 5 years of age or younger (OR, 4.29; CI_95%_, 1.17 to 15.68, *P* < 0.03) and were covered by either two antigens (*n* = 3/21) or three antigens (*n* = 18/21).

### Coverage estimation by exact matches to the vaccine peptides—no cross-protection.

The peptides used in the 4CMenB vaccine are PorA P1.4, NHBA peptide 2, fHbp peptide variant 1, and NadA peptide 3. Coverage was estimated using the more stringent criterion of 4CMenB exact vaccine peptide matches only. This estimate ignores the potential of vaccine components to stimulate cross-protective antibodies. This is an important consideration, as the level of cross-protection from vaccine-induced antibodies is lower in infants than in adults (25, 26, 32).

The overall coverage estimate was 43.8% (46/105). The proportions of coverage by age group were 42.9% (*n* = 18/42), 48% (*n* = 12/25), and 42.1% (*n* = 16/38) for the groups of those under 1 year of age, 1 to 5 years of age, and over 5 years of age, respectively. cc41/44 coverage was 88.9% (*n* = 40/45), with age group coverage levels of 90% (*n* = 18/20), 92% (*n* = 12/13), and 83% (*n* = 10/12) for the groups of those under 1 year of age, 1 to 5 years of age, and over 5 years of age, respectively.

Exact peptide coverage for all non-cc41/44 isolates (*n* = 60) was 10% (6/60). No exact peptide matches were observed in cc269 isolates.

## DISCUSSION

Globally, MATS estimates vary considerably, from 66% (CI_95%_, 46% to 78%) in Canada to 91% (CI_95%_, 72% to 96%) in the United States (33, 34). In this study, the overall 4CMenB MATS strain coverage for the RoI was estimated at 69.5% (CI_95%_, 64.8% to 84.8%). This estimate is more similar to the estimates determined for the United Kingdom (73% [CI_95%_, 57% to 87%] in 2007 to 2008 and 66% [CI_95%_, 52% to 80%] in 2014 to 2015), Spain (69% [CI_95%_, 48% to 85%]), and Austria (68% [CI_95%_, 56% to 73%), than to those determined other participating European countries where coverage point estimates are typically over 80%, including Germany (82% [CI_95%_, 69% to 92%), France (85% [CI_95%_, 69% to 93%]), Norway (85% [CI_95%_, 76% to 98%]), Italy (87% [CI_95%_, 70% to 93%]), and Greece (88% [CI_95%_, 60% to 96%]) (32, 35–37).

The levels of diversity of vaccine target peptides among the Irish MenB strains were consistent with previous descriptions of meningococcal diversity; a few variants accounted for the majority of observations, and a larger number of variants were observed at lower frequencies. The high MATS coverage for the cc41/44 isolates is also consistent with previous reports.

When the RP value determined for a particular antigen is greater than or equal to the corresponding PBT value (or if the isolate possesses a *porA* p1.4 peptide), an isolate is considered MATS positive. PBT values were established as a link to the correlate of protection in experiments using postvaccination sera taken from infants after the fourth dose at 12 months (26). This is important to consider when interpreting the present MATS coverage estimate of 61.9% (CI_95%_, 57.1% to 90.5%) in infants under 1 year of age (the target population) and in regard to those immunized at 2, 4, and 12 months of age in the RoI.

Further, infants typically produce weaker immune responses that are of shorter duration and less likely to produce a cross-protective immune response than adult vaccinees (12, 38). The difficulty in producing a cross-reactive immune response in infants has been demonstrated for fHbp (39, 40). Half of the strains studied here expressed either no antigen (30.5%) or one antigen (21%). Single-antigen coverage was mostly dependent on the level of fHbp coverage (18.1%), with minor contributions from NHBA (1.9%) and *porA* (1%).

MATS was deliberately designed with an element of conservatism. MATS RP values are based on individual antigen targets only, and any mutual cooperation between different vaccine antigens is ignored, as are any possible contributions from the OMV minor antigens, including any contribution of nonprotein antibodies. Also, NadA expression is repressed and suboptimally expressed under the *in vitro* experimental temperature condition of 37°C (31). One study which looked at a representative panel of United Kingdom MenB strains where MATS-predicted coverage was 70% (CI_95%_, 55% to 85%) reported a coverage estimate of 88% (CI_95%_, 72% to 95%) based on examination of pooled infant and adolescent using the human serum bactericidal antibody assay (hSBA) (41). The synergistic effect of antibodies binding to more than one target *in vivo* may result in enhanced bactericidal activity against a meningococcus expressing multiple vaccine targets (26).

A high proportion (27.6%, *n* = 29/105) of Irish isolates were predicted to be covered by three vaccine antigens, PorA, fHbp, and NHBA, compared to the global triple-antigen coverage of 15.7% previously reported (43). Coverage estimates could be considered more robust for these strains than for those covered by a single antigen (26). A redundancy of targets is more likely to facilitate antibody binding, increasing the likelihood of protection. The high triple-antigen coverage in the most prevalent strains (ST-154 and ST-41) is reassuring, as is the association between invasive strains harboring 3 MATS-positive peptides and infants under 1 year of age. Donnelly and colleagues reported an association between the number of vaccine targets and the level of killing; strains harboring 2 or more antigens are more likely to induce bactericidal killing (96%) than strains harboring a single antigen only (80%).

Nonrandom associations between meningococcal clonal complexes and age have been previously demonstrated (44, 45). The predominant disease-causing lineage during the studied period, cc41/44, was more likely to invade those less than 5 years of age, exhibited high exact peptide coverage (89%), and had a redundancy of MATS-positive targets.

Together, these data suggest that routine vaccination of infants during this period would have offered vaccinees protection against the most common disease-causing lineage and the two most frequently isolated disease-causing clones (ST-41 and ST-154). Nine deaths resulted from the 105 MenB IMD case episodes, six of which were predicted by MATS to be covered and might have been prevented by individual vaccination (http://www.HPSC.ie). Two of the three deaths which occurred in infants over the study period might have been prevented by 4CMenB vaccination. Those infants would have received one or two doses of 4CMenB, given their age at the time of death. There is some evidence to support the idea that partial vaccination may result in milder disease (37).

As well-engineered as the MATS *in vitro* assay is, it remains unlikely to be wholly representative of the more complex *in vivo* conditions of bacterial invasion. Individual immune responses among vaccinees exhibit variation. When Bexsero was used to control a MenB cc41/44 meningococcal outbreak that was caused by a clone expressing both fHbp and NHBA peptides which matched those in the vaccine, 33.9% of 499 adult vaccinees failed to generate a detectable protective antibody response against the outbreak strain 8 weeks after the second dose (46).

Bexsero has been licensed and deployed on the basis of serological and immunological criteria only. These methods are substitutes for measuring vaccine efficacy directly from protection studies, where a reduction in disease incidence in a sufficiently large population may be attributed to the vaccine. Data from the United Kingdom national immunization program have provided the first evidence of the effectiveness of Bexsero at the population level, where disease is caused by many genetically variable strains (17). Parikh et al. used the screening method to compare vaccine-eligible and vaccine-ineligible cohorts, 10 months following the introduction of the vaccine into the routine infant schedule, and observed an 83% level (CI_95%_, 24% to 95%) of two-dose vaccine effectiveness against all MenB cases (17). A 50% (CI_95%_, 0.36 to 0.71, *P* = 0.001) incidence rate ratio (IRR) reduction was observed in the vaccine-eligible cohort compared to the estimated number of cases in infants of the same age for the 4 years prior to the vaccine’s introduction. A reduction of 36% is reported following correction for a declining SgB incidence (CI_95%_, 0.08 to 0.55, *P* = 0.015). These data provide the first evidence of real-world vaccine effectiveness. Although the precise level of effectiveness is unclear as yet, these data are particularly comforting, as the small population and the incidence of MenB disease in the RoI will hinder similar measurements of the vaccine’s effectiveness.

Predicted coverage of Bexsero varies throughout Europe as a consequence of the diversity of the meningococcal population, which contains elements that are stable and elements that are subject to temporal and geographic flux (4, 47). Researchers in the United Kingdom have reported MATS data from two different periods and observed a coverage change from the 2007–2008 estimate of 73% (CI_95%_, 57% to 87%) to the more recent estimate of 66% (CI_95%_, 52% to 80%) for the 2014–2016 period. The changing nature of meningococcal populations presents a challenge to predicting the impact of subcapsular vaccines both ahead of implementation and postdeployment. The validation of MATS estimations from national immunization programs such as those in the United Kingdom may enable the use of MATS to monitor the effectiveness of the vaccine in the RoI and elsewhere. While whole-genome sequencing (WGS) does not directly determine the extent of antibody immunological cross-reactivity, as the number of isolates that have generated MATS data and WGS data increases, it may be possible to refine a model to predict the MATS result from genotype data (48, 49). This is important, since only a minority of IMD cases are culture positive.

Continuous invasive strain characterization, including 4CMenB vaccine peptide sequence data, will be essential to monitor changes in circulating meningococci and to recognize the emergence and expansion of new clones, especially those with novel 4CMenB peptides or strains exhibiting nonsusceptible vaccine peptide profiles, and to investigate potential vaccine failures.

## MATERIALS AND METHODS

### Isolates.

Cultured invasive meningococcal isolates are sent to the Irish Meningitis and Sepsis Reference Laboratory (IMSRL) for characterization. A total of 326 real-time PCR-confirmed MenB IMD cases occurred during the 2009–2010 to 2012–2013 epidemiological years. Of these, 105 (32.2%) were culture positive, and all 105 invasive isolates were included in the study for MATS testing.

### Sequencing.

The *porA* variable regions were determined by Sanger sequencing, and the data were then used to deduce the PorA VR peptides using minor adaptions of previously defined protocol (50) and were curated using the PubMLST Neisseria website (http://www.pubmlst.org/neisseria) (51). Purified genomic DNA for all isolates was prepared using a Wizard kit (Promega) in accordance with the manufacturer’s instructions. DNA quantity and quality were assessed using a Qubit fluorometer (Life Technologies, Inc.) and standard 1% agarose gel electrophoresis, respectively. Genomic DNA was sequenced on an Illumina HiSeq sequencer at the Oxford Genomics Center, Wellcome Trust Centre for Human Genetics, University of Oxford, United Kingdom. The short-read sequences were assembled de novo using the VelvetOptimiser algorithm (52) as part of an in-house-validated pipeline developed in Oxford and were then added to the Bacterial Isolate Genome Sequence Database (BIGSdb) on the PubMLST Neisseria website (51). Genomes were automatically scanned against alleles previously defined in the sequence definition database. MLST data and 4CMenB target peptides were extracted using the “MLST” and “antigen” options with the “scheme” tool in BIGSdb (53).

### MATS.

MATS assays were performed at PHE-MRU, Manchester, United Kingdom (29). MATS ELISA RP values were determined independently for each antigen by comparisons with reference strains expressing each vaccine antigen (26). An isolate was predicted to be covered if the PorA VR2 P1.4 peptide was present and/or if an RP value greater than its MATS-PBT (0.021 for fHbp, 0.294 for NHBA, and 0.009 for NadA) was obtained for at least one antigen, as described previously (26). The PBT is the minimum RP value that is predictive of whether a MenB isolate would be susceptible to killing in the human serum bactericidal antibody assay (hSBA) by 4CMenB-elicited antibodies. The 95% confidence intervals for the positive PBT thresholds were derived as described previously (29). The threshold values established are as follows: 0.014 to 0.031 for fHbp, 0.169 to 0.511 for NHBA, and 0.004 to 0.019 for NadA.

### Statistical analysis.

As described in the MATS interlaboratory standardization study (29), empirical estimates of the 95% CIs for the positive bactericidal thresholds were derived with a log-normal approximation based on the overall assay reproducibility (0.014 to 0.031 for fHbp, 0.169 to 0.511 for NHBA, and 0.004 to 0.019 for NadA). These values were used to define the 95% CIs of strain coverage. No CIs are calculated for coverage by the PorA antigen, as the typing was performed genotypically (VR2 = P1.4).

All statistical evaluations were performed using R statistical software (http://www.r-project.org) version 3.2.3.
