# Supplementary figures and images for: Potential Coverage of the 4CMenB Vaccine against Invasive Serogroup B Neisseria meningitidis Isolated from 2009 to 2013 in the Republic of Ireland
Source: mSphere. 2018 Aug 22;3(4):e00196-18. doi: 10.1128/mSphere.00196-18 (PMC6106058; doi:10.1128/mSphere.00196-18)

Fig. S1. Relative potency values of the most frequently observed NHBA peptides.

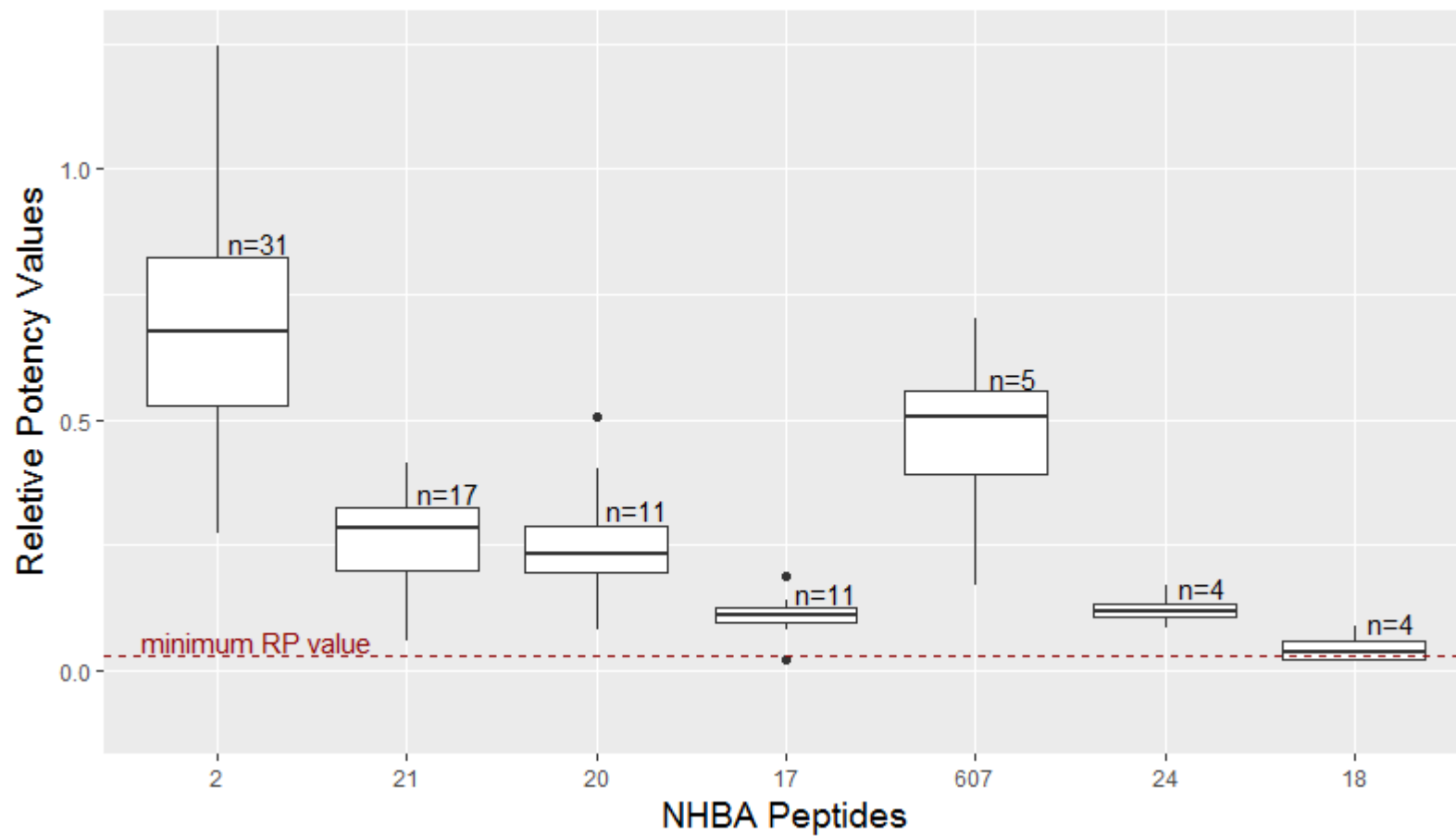

Supplement: FIG S1 [file sph004182630sf1.pdf]

Fig. S2. Relative potency values of the most frequently observed fHbp peptides.

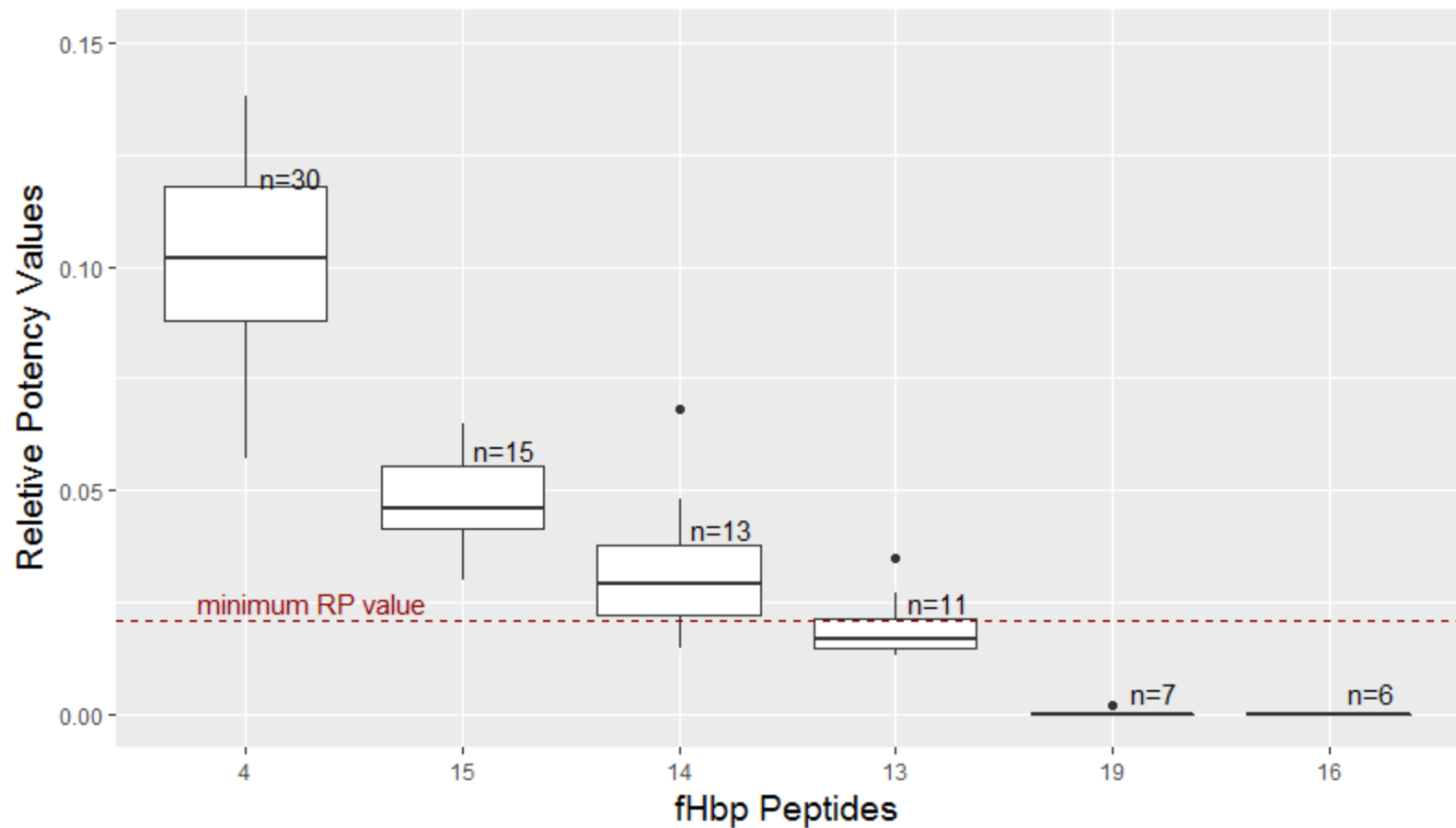

Supplement: FIG S2 [file sph004182630sf2.pdf]
